# Benign prostatic hyperplasia and metabolic syndrome; prevalence and association: a cross-sectional study in Syria

**DOI:** 10.1186/s12894-023-01365-9

**Published:** 2023-11-16

**Authors:** Mohanad Daher, Tareq Saqer, Mahmoud Jabr, Samaher Al-Mousa

**Affiliations:** 1 Faculty of medicine, Damascus University, Damascus, Syria; 2 Faculty of medicine, Al-Baath University, Homs, Syria; 3 Department of Rheumatology, Tishreen Military Hospital, Damascus, Syria

**Keywords:** Lower urinary tract symptoms, Male, Metabolic syndrome, Prostate, Prostatic hyperplasia

## Abstract

**Background:**

Benign Prostatic Hyperplasia (BPH) is considered the most common cause of lower urinary tract symptoms in men aged 40 years and older. BPH is related to sex steroids, but there are increasing studies investigating the relationship between the urinary symptoms and the metabolic syndrome. They still have inconsistent results; some reported a significant positive association, while others found no significant association. In this study, we aim to assess the prevalence rate of metabolic syndrome in BPH patients and whether there is an association between symptoms linked to BPH and metabolic syndrome in the Syrian community.

**Methods:**

The participants of this observational cross-sectional study were benign prostatic hyperplasia patients aged 40-year-old and older from Homs, Syria. An interview questionnaire was performed to collect data from all patients who visited the urology clinic of Homs Military Hospital in the period of January 10 to March 10, 2023. We used the International Prostate Symptom Score (IPSS) to assess the urinary symptoms, and we used the US National Cholesterol Education Program Adult Treatment Panel (NCEP ATPIII) criteria to define the metabolic syndrome.

**Results:**

The final sample size was 426 patients. The overall prevalence of metabolic syndrome was 46.2%. Patients with metabolic syndrome had higher International Prostate Symptom Score compared to patients without metabolic syndrome (21 vs. 18, *P* < 0.001), and 59.3% of patients with metabolic syndrome suffered from severe symptoms compared to 36.2% of patients without metabolic syndrome who suffered from severe lower urinary tract symptoms (*P* < 0.001). There was a positive association between (waist circumference, diabetes, triglycerides) (*P* < 0.001), HDL (*P* = 0.014) and higher International Prostate Symptom Score. However, there was no statistically significant association between blood pressure and International Prostate Symptom Score (*P* = 0.879).

**Conclusion:**

Our results showed that patients with metabolic syndrome had a higher International Prostate Symptom Score. This idea should be used to design a new benign prostatic hyperplasia/lower urinary tract symptoms treatment.

## Background

### Lower urinary tract symptoms

“Lower urinary tract symptoms” (LUTS) is a general term. It has been used to refer to any combination of urinary symptoms or, more specifically, the symptoms most frequently linked to an overactive bladder (frequency, urgency, and nocturia). An international consensus conference defined LUTS to include signs of voiding and/or storage problems that are typical in aged males [1], such as hesitancy, a weak or inconsistent stream, straining, prolonged peeing, the sensation that the bladder isn’t emptying completely, dribbling, frequency, urgency, urge incontinence, and nocturia [2].

### Benign prostatic hyperplasia

Benign prostatic hyperplasia (BPH) is the leading cause of LUTS [3]. BPH is a condition in men in which the prostate gland is enlarged but not cancerous. As the prostate enlarges, the gland presses against and pinches the urethra, causing many of the problems associated with benign prostatic hyperplasia [4]. Benign prostatic hyperplasia is the most common prostate problem for men older than 50 [4], about 15 million American men are affected according to Lee et al. [5] and 22.7% of Chinese men aged 70 years and older are affected [6].

### Metabolic syndrome

The complicated illness known as metabolic syndrome (MetS), which has a large socioeconomic cost, is considered as an epidemic on a global scale [7]. MetS is characterized by a collection of linked factors that significantly raise the risk of coronary heart disease. Its main features are dyslipidemia (elevated triglycerides and low high-density lipoproteins (HDL)), elevation of arterial blood pressure (BP), and dysregulated glucose homeostasis; however, abdominal obesity and/or insulin resistance (IR) have received growing attention as the syndrome’s core manifestations [7]. There are almost four definitions of metabolic syndrome: the World Health Organization (WHO), the International Diabetes Foundation (IDF), the National Cholesterol Education Program Adult Treatment Panel III (NCEP ATPIII), and the European Group for the Study of Insulin Resistance (EGIR). In our study, we used NCEP ATPIII criteria [8].

### Lower urinary tract symptoms and metabolic syndrome

The cause of benign prostatic hyperplasia is not well understood, but sex steroids, such as estrogen or testosterone, are still the main known factors promoting gland growth [9]. Many studies in different countries have investigated the possible association between LUTS and MetS, as having metabolic syndrome may increase the severity of lower urinary tract symptoms, but they still have inconsistent results; some reported a significant positive association [10–12], others found no significant association [13, 14]. In this study, which is the first of its kind in Syria, the prevalence of metabolic syndrome will be evaluated among a sample of benign prostatic hyperplasia patients in one Syrian city, and the severity of lower urinary tract symptoms will be evaluated and compared with non-metabolic syndrome patients to find out whether metabolic syndrome is associated with increasing the severity of LUTS linked to BPH or not.

## Materials and methods

### Study design, participants, setting, duration and ethical considerations

We conducted an observational cross-sectional study at the urology clinic of Homs Military Hospital, Homs, Syria, from January 10 to March 10, 2023. The institutional research ethics board’s approval was obtained from the Homs Military Hospital ethical board before performing any study procedure (approval no. 56,851). The participants were 40-year-old and older benign prostatic hyperplasia (BPH) patients who visited the clinic during the study period and were diagnosed according to a prior prostate biopsy, taking only an alpha blocker as a medication or on drug treatment for one of the metabolic syndrome components as NCEP ATPIII defined the metabolic syndrome. Informed consent was obtained from all the participants and their identities are kept confidential.

### Sample size calculation

Assuming a prevalence rate of metabolic syndrome in BPH patients of 16.6% as reported in a previous study by (Ohgaki et al. 2011) [15], this study required 426 participants to detect a similar prevalence rate with a 5% deviation and 95% confidence level. The sample size was calculated using the OpenEpi online calculator according to the calculation equation mentioned by Charan et al. [16]. The sampling method was non-randomized and non-intentional (convenience sampling).

### Variables and data collection

We performed an interview questionnaire to collect the following data: age, body mass index (BMI), marital status, smoking, and the International Prostate Symptom Score (IPSS). We used IPSS to assess the lower urinary tract symptoms. The IPSS items are: feeling of incomplete emptying, increased daytime frequency, intermittency, urgency, slow stream, straining, and nocturia over the past month, and the score ranges from mild (0–7) to moderate [8–19] to severe (20–35), the quality of life due to urinary symptoms is considered an additional item, but it is not used in the score calculation [17]. We used the US National Cholesterol Education Program Adult Treatment Panel (NCEP-ATPIII) [8] criteria to define the metabolic syndrome as having any three or more of the five components (blood pressure: systolic blood pressure (SBP) ≥ 130 mmHg or/and diastolic blood pressure (DBP) ≥ 85 mmHg or on antihypertensive drug treatment, fasting blood glucose: ≥ 110 mg/dl or on drug treatment for elevated glucose, waist circumference: ≥ 102 cm, high-density lipoprotein (HDL): ≤ 40 mg/dl and triglycerides (TG): ≥ 150 mg/dl or on drug treatment for elevated triglycerides). We assessed the metabolic syndrome by collecting 4 ml of venous blood from each respondent to test blood biomarkers: HDL, TG, and fasting glucose. We measured waist circumference by asking the patients to stand still and take a normal breath, then exhale and hold their breath at the end of their exhalation. Then we put the measuring tape at the level of the navel. We measured each participant’s blood pressure three times, then took the average of the three measurements. Prostate volume was measured using transabdominal ultrasound by a urology resident.

### Statistical analysis

We analyzed the data using SPSS v25 statistics software for Windows. In all cases, a p value < 0.05 was considered statistically significant. Categorical data on descriptive analysis was presented in absolute and relative frequency, average, and grouped by MetS (with versus without MetS). Continuous data was presented as median and interquartile range; it was not normally distributed after using the Kolmogorov-Smirnov test to test normality. The associations between the different socio-demographic variables and each of the sub-groups by MetS were obtained using the Chi-squared test for categorical variables and the Mann-Whitney test for continuous variables. We used the Mann-Whitney test to compare patients with or without MetS in IPSS score and prostate volume. We used the Kruskal-Wallis test to find out if there is an association between prostate volume and age on the one hand and prostate volume and symptoms on the other.

## Results

The final sample size was 426 patients. The overall prevalence rate of metabolic syndrome was 46.2%. The median age was 64 years. The median BMI was 28 kg/m^2^ and 13.6% of men were obese (BMI ≥ 30 kg/m^2^). The patients’ characteristics are shown in (Table 1), including the frequency of each metabolic syndrome component. Elevated blood pressure, elevated fasting glucose, and elevated waist circumference were the three most frequently reported metabolic syndrome criteria. The median IPSS score was 19, and 46.9% of patients had severe BPH-related lower urinary tract symptoms. The median prostate volume was 40 cc, with 21.1% of patients having a high-volume prostate (more than 60 cc). The association between each variable and metabolic syndrome is shown in (Table 2). There was no difference in prostate volume between patients with or without metabolic syndrome (40 vs. 40, *P* = 0.743). Patients with metabolic syndrome had a higher IPSS score compared to patients without metabolic syndrome (21 vs. 18, *P* < 0.001), and 59.3% of patients with metabolic syndrome suffered from severe LUTS compared to 36.2% of patients without metabolic syndrome who suffered from severe LUTS (*P* < 0.001). We discovered a statistically significant positive relationship between metabolic syndrome and three individual IPSS questions: Q2 (*P* = 0.013), Q3 (*P* = 0.006), and Q7 (*P* < 0.001). The association between each individual component of the metabolic syndrome and LUTS is shown in (Table 3). There was a positive association between (waist circumference, diabetes, triglycerides) (*P* < 0.001), HDL (*P* = 0.014) and a higher IPSS score. However, there was no statistically significant association between blood pressure and IPSS score (*P* = 0.879). We found a statistically significant difference between prostate volumes according to age, as older ages were associated with larger volumes (*P* < 0.001), and we found a statistically significant difference between prostate volumes according to LUTS severity, as patients who suffered from severe symptoms had the higher volume (*P* < 0.001). There was no statistically significant association between prostate volumes and BMI (*P* = 0.728), but there was a statistically significant difference between BMI categories (< 25, 25–30, and > 30 kg/m2) in IPSS score medians (18, 20.5, and 21), respectively (*P* < 0.001). Also, 58.6% of obese patients had severe symptoms (IPSS score ≥ 20), whereas only 33.6% of normal BMI patients < 25 had severe symptoms (*P* = 0.001).

**Table 1:** Characteristics of the study population

	Total sample *N* = 426
Age	64 (58–70)*
Age (categories)	
40–50	20 (4.7%)
51–60	127 (29.8%)
61–70	188 (44.1%)
71–80	75 (17.6%)
> 80	16 (3.8%)
BMI^a^	28 (25–30)*
BMI (categories)	
< 25	134 (31.5%)
25–30	234 (54.9%)
> 30	58 (13.6%)
Marital status	
Married	424 (99.5%)
Not married	2 (0.5%)
Smoking	
Yes	254 (59.6%)
No	172 (40.4%)
IPSS^b^	19 (14–24)*
IPSS severity	
Mild	52 (12.2%)
Moderate	174 (40.8%)
Severe	200 (46.9%)
Blood pressure: SBP^c^ ≥130 mmHg or/and DBP^d^ ≥ 85 mmHg or on antihypertensive drug treatment	
Yes	263 (61.7%)
No	163 (38.3%)
Fasting blood glucose: ≥ 110 mg/dl or on drug treatment for elevated glucose	
Yes	208 (48.8%)
No	218 (51.2%)
Waist circumference: ≥ 102 cm	
Yes	129 (30.3%)
No	297 (69.7%)
HDL^e^: ≤ 40 mg/dl	
Yes	280 (65.7%)
No	146 (34.3%)
Triglycerides: ≥ 150 mg/dl or on drug treatment for elevated triglyceride	
Yes	271 (63.6%)
No	155 (36.4%)
Metabolic syndrome	
Yes	197 (46.2%)
No	229 (53.8%)
Prostate Volume	40 (30–55)*

*not normally distributed a: body mass index b: international prostate symptom score

**c: systolic blood pressure d: diastolic blood pressure e: high-density lipoprotein**

**Table 2:** Association between variables and metabolic syndrome

	With MetS^a^*N* = 197 (46.2%)	Without MetS *N* = 229 (53.8%)	*P* value
Age	65	63	0.007*
BMI	28	27	< 0.001*
Smoking			0.037•
Yes	128 (30%)	126 (29.6%)	
No	69 (16.2%)	103 (24.2%)	
IPSS score	21	18	< 0.001*
IPSS severity			< 0.001•
Mild (0–7)	16 (3.8%)	36 (8.5%)	
Moderate (8–19)	64 (15%)	110 (25.8%)	
Severe (20–35)	117 (27.5%)	83 (19.5%)	
Prostate volume	40	40	0.743*
Q1			0.423•
0	101 (23.7%)	122 (28.6%)	
1–2	6 (1.4%)	12 (2.8%)	
3–5	90 (21.1%)	95 (22.3%)	
Q2			0.013•
0	78 (18.3%)	122 (28.6%)	
1–2	18 (4.2%)	21 (4.9%)	
3–5	101 (23.7%)	86 (20.2%)	
Q3			0.006•
0	30 (7%)	51 (12%)	
1–2	2 (0.5%)	12 (2.8%)	
3–5	165 (38.7%)	166 (39%)	
Q4			0.068•
0	57 (13.4%)	90 (21.1%)	
1–2	14 (3.3%)	17 (4%)	
3–5	126 (29.6%)	122 (28.6%)	
Q5			0.065•
0	20 (4.7%)	40 (9.4%)	
1–2	6 (1.4%)	10 (2.3%)	
3–5	171 (40.1%)	179 (42%)	
Q6			0.955•
0	81 (19%)	96 (22.5%)	
1–2	17 (4%)	18 (4.2%)	
3–5	99 (23.2%)	115 (27%)	
Q7			< 0.001•
0	4 (0.9%)	12 (2.8%)	
1–2	37 (8.7%)	77 (18.1%)	
3–5	156 (36.6%)	140 (32.9%)	

*Mann-Whitney test •Chi-squared test a: metabolic syndrome

**Table 3:** Association between each metabolic syndrome component and LUTS.

	IPSS severity			*P* value
	Mild (0–7)	Moderate (8–19)	Severe (20–35)	
**Waist circumference (≥ 102 cm)**				< 0.001*
Yes: 297 (69.7%)	29 (6.8%)	109 (25.6%)	159 (37.3%)	
No: 129 (30.3%)	23 (5.4%)	65 (15.3%)	41 (9.6%)	
**Blood pressure (SBP ≥ 130 mmHg or/and DBP ≥ 85 mmHg)**				0.879*
Yes: 263 (61.7%)	33 (7.7%)	105 (24.6%)	125 (29.3%)	
No: 163 (38.3%)	19 (4.5%)	69 (16.2%)	75 (17.6%)	
**Fasting blood glucose (≥ 110 mg/dl)**				< 0.001*
Yes: 218 (51.2%)	36 (8.5%)	68 (16%)	114 (26.8%)	
No: 208 (48.8%)	16 (3.8%)	106 (24.9%)	86 (20.2%)	
**HDL (≤ 40 mg/dl)**				0.014*
Yes: 146 (34.3%)	10 (2.3%)	56 (13.1%)	80 (18.8%)	
No: 280 (65.7%)	42 (9.9%)	118 (27.7%)	120 (28.2%)	
**Triglycerides (≥ 150 mg/dl)**				< 0.001*
Yes: 155 (36.4%)	12 (2.8%)	51 (12%)	92 (21.6%)	
No: 271 (63.6%)	40 (9.4%)	123 (28.9%)	108 (25.4%)	

## Discussion

In this cross-sectional study of 426 Syrian men diagnosed with benign prostatic hyperplasia, we investigated the relationship between MetS with its components and BPH. We found that the prevalence rate of MetS in BPH patients was 46.2% identified by NCEP-ATP III criteria, which is close to the overall prevalence rate in all populations, and that was roughly compatible with other studies in different countries: 41.6% in Iran [18], 33.6% in Palestine [19], 33.9% in Turkey [20] and 51.5% in France [21], one of the crucial elements that needs special consideration is that there are many definitions of MetS, this may explain the disparity in prevalence rates between studies. The present study found no difference in prostate volume between patients with and without metabolic syndrome, while other observational studies found a link between metabolic syndrome and prostate volume [22, 23], also a recent systematic review reported that patients with MetS had higher annual prostate growth rate [24]. Our study suggested that there is an association between the severity of lower urinary tract symptoms and metabolic syndrome, where patients with metabolic syndrome had a higher IPSS score compared to those without metabolic syndrome, and this result was indicated in previous studies, Yongqiang Fu et al. [25] suggested that MetS, in particular, diabetes mellitus and hypertension, may accelerate the clinical progression of BPH. Pashootan et al. [21] found a significant relationship between LUTS linked to benign prostatic hyperplasia and metabolic syndrome, in terms of frequency and severity. However, some studies in other countries reported no significant association between LUTS and MetS, Gupta et al. [14] showed no relationship between BPH and metabolic syndrome, weight, body mass index, or lipid level. Park et al. [13] found no statistically significant differences in voiding symptoms between the metabolic and non-metabolic groups. Ohgaki et al. [15] suggested that LUTS is associated with aging, regardless of the presence of the metabolic syndrome. These disparities in results between previous studies could be attributed to a variety of factors, including differences in lifestyle, economic status, sample size, study design, and sample population. Furthermore, we found a positive association between each individual metabolic syndrome component (diabetes, HDL, triglycerides, and waist circumference) and higher IPSS, Xiong et al. [10] found similar results. Ferreira et al. [12] reported a positive association between diabetes mellitus and the increase of the LUTS, Lee et al. [11] found that increased waist circumference is associated with worsened voiding symptoms, even bariatric surgery may be an effective intervention for improving the lower urinary tract symptoms in obese male patients, as mentioned by Liu et al. [26] However, we found no statistically significant association between blood pressure and IPSS score as Xiong et al. [10] found. The precise mechanism connecting BPH and metabolic syndrome is still unclear, maybe explained by hyperinsulinemia [27] or increased oxidative stress in accumulated fat in obese patients, this is what causes DNA damage in the prostate promoting epithelial proliferation, stromal thickening and fibrosis, which interferes with the pathophysiology of BPH [28]. Also, the DNA damage caused by over oxidative stress promotes an inflammatory response, and in surgical specimens, there are clear correlations between BPH and histological inflammation, with the intensity of the inflammation correlating with the size of the enlarged prostate and the area of BPH [29, 30]. The present study showed a statistically significant difference between prostate volumes according to age and LUTS, as older ages and those who suffered from severe symptoms had the higher volumes, and that was compatible with Zhang et al. [31] study. The following limitations may have an impact on how reliable our research is. First, because only Syrian BPH patients were included in this study, the results cannot be applied to other populations. Second, as a cross-sectional study, the current investigation did not take time into account. Finally, while some possible confounders were taken into account in the analysis, others, such as income and educational status were not, which could have introduced bias into the research.

## Conclusion

the present study is the first population-based study in Syria to investigate the relationship between the presence of metabolic syndrome and LUTS. Our results showed that patients with MetS had a higher IPSS score. We suggest that this result be taken into consideration to design a new BPH/LUTS treatment, especially when symptoms do not respond to the known drug therapy. So, lifestyle changes, such as physical exercises, weight loss, quitting smoking, and even lipid-lowering drugs, may play an important role in relieving severe BPH symptoms.

8 **Tables**:

## Data Availability

The datasets used and/or analyzed during the current study are available from the corresponding author on reasonable request.

## List of Abbreviations

BPH: Benign prostatic hyperplasia
LUTS: Lower urinary tract symptoms
IPSS: International Prostate Symptom Score
BMI: Body mass index
NCEP-ATPIII: US National Cholesterol Education Program Adult Treatment Panel
MetS: Metabolic syndrome
SBP: Systolic blood pressure
DBP: Diastolic blood pressure
HDL: High-density lipoprotein
TG: Triglycerides
